# Transcranial Direct Current Stimulation of the Occipital Cortex in Medication Overuse Headache: A Pilot Randomized Controlled Cross-Over Study

**DOI:** 10.3390/jcm9041075

**Published:** 2020-04-10

**Authors:** Anthony G. Mansour, Rechdi Ahdab, Georges Khazen, Christelle El-Khoury, Toni M. Sabbouh, Maher Salem, Wissam Yamak, Moussa A. Chalah, Samar S. Ayache, Naji Riachi

**Affiliations:** 1 Department of Internal Medicine, the Ohio State University, Columbus, OH 43210, USA; anthony.gmansour@gmail.com; 2 Gilbert and Rose Mary Chagoury School of Medicine School of Medicine, Lebanese American University, Byblos 4504, Lebanon; chadahdab@gmail.com (R.A.); gkhazen@lau.edu.lb (G.K.); christelle.elkhoury@lau.edu (C.E.-K.); Maher.Salem@lau.edu (M.S.); wissam.yamak@lau.edu.lb (W.Y.); 3 Division of Neurology, Hamidy Medical Center, Tripoli 1300, Lebanon; 4 Division of Neurology, Lebanese American University Medical Center Rizk Hospital, Beirut 113288, Lebanon; 5 Computer Science and Mathematics Department, Lebanese American University, Byblos 4504, Lebanon; 6 Division of Family Medicine, Lebanese American University Medical Center, Beirut 113288, Lebanon; 7 Department of Internal Medicine, Morristown Medical Center, Morristown, NJ 07960, USA; toni.sabbouh@lau.edu; 8 Service de Physiologie-Explorations Fonctionnelles, Hôpital Henri Mondor, Assistance Publique- Hôpitaux de Paris, 94010 Créteil, France; moussachalah@gmail.com (M.A.C.); samarayache@gmail.com (S.S.A.); 9 EA 4391, Excitabilité Nerveuse et Thérapeutique, Université Paris-Est-Créteil, 94010 Créteil, France

**Keywords:** cathodal occipital tDCS, anodal prefrontal tDCS, medication overuse headaches

## Abstract

Background: Medication overuse headache (MOH) is a chronic pain syndrome that arises from the frequent use of acute antimigraine drugs. Transcranial direct current stimulation (tDCS) is a non-invasive brain stimulation technique with a possible therapeutic effect in this particular context. Methods: This was a randomized, sham-controlled, cross-over study. Eighteen patients with MOH (17 women, age range: 20–38 years) received three sets of three consecutive daily sessions of tDCS: anodal tDCS over the prefrontal cortex, cathodal tDCS over the occipital cortex ipsilateral to the dominant side of migraine pain, and sham. The order in which the tDCS blocks were delivered was randomly defined based on a 1:1:1 ratio. Patients filled in a migraine diary that allowed recording of the pain intensity (visual analogue scale) and the daily consumption of analgesic pills from one week before to two weeks after each condition. Results: Both prefrontal and occipital tDCS lowered the total number of migraine days and the number of severe migraine days per week at week 1, but only the effects of occipital tDCS on these two outcomes lasted until week 2. Only occipital tDCS decreased the daily analgesic pills consumption, at weeks 1 and 2. Conclusion: Three consecutive days of cathodal occipital tDCS appear to improve the clinical outcomes in patients with MOH.

## 1. Introduction

Medication overuse headache (MOH) is a chronic pain syndrome that arises from the frequent use of acute antimigraine drugs. This condition affects around 60 million individuals worldwide and has a one-year prevalence of 1–2% in the general population [1]. The International Headache Society defines MOH as a headache caused by regular overuse of acute headache treatments in a patient with pre-existing primary headache disorder [2]. It has a frequency of 15 or more days per month for more than 3 months and is associated with medication use for a period equal or superior to 10–15 days per month [2]. MOH is considered a major cause of disability and can drastically affect the quality of life [1].

MOH appears to be more frequent among women, as well as among individuals with chronic pain conditions, comorbid anxiety or depression [1,3,4]. Other risk factors include substance-related disorder spectrum, and family history of MOH or other substance use disorders (i.e., illicit drugs and alcohol) [5,6,7]. Patient education and withdrawal of the overused medications are integral parts of the treatment strategy [1]. Treatment of comorbid psychiatric and substance abuse disorders and management of the background primary headache by using adequate preventive drug therapy are also recommended, although high-quality data to support these approaches are scarce [1].

Various non-invasive neuromodulatory techniques have been studied in migraine with variable results [8]. One approach of particular interest is the transcranial direct current stimulation (tDCS), a widely available technique with an excellent safety profile, good practicality, and relative low cost [9,10]. tDCS consists of applying a weak electric current (i.e., 1–2 mA) for a short duration (i.e., 10–30 min) over specific cortical sites via two electrodes (i.e., cathode and anode) connected to a battery-driven stimulator [9]. Based on physiological studies targeting the motor cortex, anodal and cathodal tDCS seem to respectively result in a shift of the resting membrane potential toward a depolarization and hyperpolarization [9].

A growing body of evidence supports the efficacy of tDCS in preventing migraine attacks [11,12,13,14,15,16,17,18,19]. On the one hand, tDCS in this context was proven efficacious when cathodal stimulation is applied to the occipital (visual) cortex [11,14,15,16,19]. The rationale for this choice of target is the now well-established functional and morphological abnormalities that manifest interictally in patients with migraine [20]. These include abnormal visual processing [21,22], visual cortex hypermetabolism [23,24], and reduced cortical and cerebellar inhibition [25,26]. In addition, migraine triggers (e.g., stress, fatigue, hormones, sleep deprivation, weather, and seasonal variation) seem to act on a pre-existing hyperexcitable cortex and induce a cortical spreading depression, which has been widely described during migraine attacks [27,28,29].

Conversely, anodal tDCS of the left dorsolateral prefrontal cortex (DLPFC) has also been proposed to modulate migraine [17]. The DLPFC is implicated in several cognitive, emotional, and behavioral processes, including emotion processing and pain control, and has been targeted by tDCS in several clinical conditions, including substance use disorders [9]. Interestingly, MOH and substance dependence share some common neurobiological pathways (such as orbitofrontal cortex dysfunction), and MOH has been shown to be associated with substance-related disorder spectrum, especially overusing psychoactive substances [7,30]. Stimulation of the left DLPFC has shown some positive results on craving in various types of addiction such as nicotine, alcohol, cocaine, and marijuana [31]. However, it remains to be proven whether this intervention is efficacious in treating migraine patients with medication abuse.

The present report presents a randomized, double-blind, sham-controlled pilot study designed to investigate the efficacy of occipital and prefrontal tDCS in the treatment of MOH.

## 2. Materials and Methods

### 2.1. Study Population

Recruitment took place at the Hamidy Medical Center, North Lebanon. Patients were recruited if they were between 18 and 60 years of age, with migraine as the pre-existing headache disorder, and a history of medication overuse headaches (i.e., International Headache Society 3rd edition criteria [2]). Patients under preventive migraine treatment could take part in the study if they had a stable dosage in the last three months and did not anticipate modifying it in the upcoming two months. Patients were not included if they had any of the following: (i) previous tDCS exposure, (ii) substance use disorder, (iii) history of seizures, pregnancy, lactation, cardiac pacemakers, scalp or head metallic hardware (e.g., surgical clips), (iv) head and neck botulinum toxin procedures in the last six months, (v) brain surgery or tumors, or (vi) failure to complete the migraine diary at baseline (i.e., less than 90% complete).

All patients voluntarily gave their written informed consent before enrollment. The local institutional review board of Hamidy Medical Center approved the study (approval number HMC#5117, date of approval 21 January 2017) which was conducted in conformity with the 1964 Declaration of Helsinki and its later amendments.

### 2.2. Study Design

This pilot study included three stimulation conditions: anodal tDCS over the prefrontal cortex, and cathodal and sham tDCS over the occipital cortex. It had a double blind, sham-controlled, and cross-over design; blinding concerned patients and investigators.

Each patient underwent the three conditions in a randomized manner (randomized in a 1:1:1 ratio). Block randomization was used to assign patients into each of the 6 groups. Each condition included a screening visit, a pre-interventional period (i.e., one week), a stimulation block lasting three days, and a post-interventional period (i.e., two weeks). The latter was followed by a one-week washout interval after which the patients underwent the next intervention.

During the screening visit, patients were given instructions to complete the migraine diary on a daily basis during the pre- and post-interventional periods (one week before and two weeks after the block). The diary included a visual analogue scale (VAS) that accounted for headache intensity and frequency, and entries on the type and number of acutely consumed analgesic pills. At the end of the pre- and post-interventional periods of each condition, patients were seen in order to verify the proper completeness of the diary, provide them with a new one, and document any side effects or specific concerns.

### 2.3. Study Endpoints

The primary endpoint was the mean variation in the number of migraine days per week comparing the baseline pre-interventional period to the 14-day post-interventional period (week 1 and week 2 (W1 and W2)). The secondary endpoint was the mean reduction from baseline in analgesic pills consumption over W1 and W2.

### 2.4. Transcranial Direct Current Stimulation

**Figure 1:**
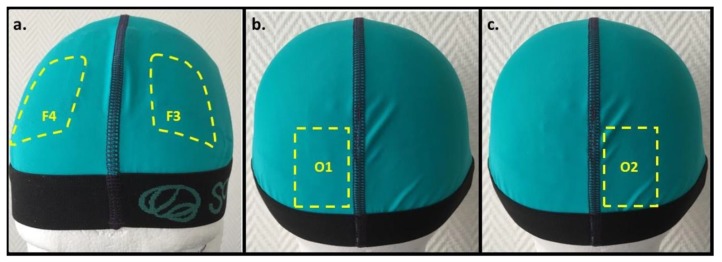
The transcranial direct current stimulation (tDCS) setup with electrodes positioned according to the 10–20 electroencephalography system. (**a**) Prefrontal tDCS: the yellow marked areas correspond to the anode and cathode position over the left (F3) and right (F4) dorsolateral prefrontal cortices, respectively. (**b**,**c**) Occipital tDCS: the yellow marked areas illustrate the cathode position over the left (O1) or right (O2) occipital cortex; the anode is placed over the contralateral supraorbital area (not shown).

tDCS sessions were performed by a trained nurse. Stimulation occurred while the patient was sitting in a comfortable chair and at rest. A Sooma tDCS^TM^ device (Sooma Oy, Helsinki, Finland) was used to deliver a weak direct current (i.e., 2 mA) via a pair of saline-soaked sponge electrodes (35 cm^2^). We referred to the 10–20 electroencephalographic system of electrode positioning. For occipital and sham tDCS, the cathode was positioned over the occipital cortex ipsilateral to the dominant side of the migraine pain (O1 or O2), and the anode was positioned over the contralateral supraorbital region (Figure 1). For prefrontal tDCS, the anode and cathode were respectively positioned over left and right DLPFC (F3 and F4). Real tDCS sessions (occipital and prefrontal) lasted 20 min each. The sham condition implied 20 s of ramping up at a velocity of 0.1 mA/s, after which the current ramped down and phased off automatically. The adopted stimulation parameters were based on a previous study undertaken on patients with episodic migraine [19].

### 2.5. Statistical Analysis

R statistical Software (version 3.5.3, the R Foundation for Statistical Computing, Vienna, Austria) was used to perform the statistical analysis. The following output measures were analyzed and compared between the prefrontal, occipital, and sham conditions. These include (i) the number of migraine days per week, (ii) the number of severe migraine days per week, and (iii) the number of consumed pills per day. The migraine days per week were computed based on VAS scores. A migraine day was counted if the VAS score was ≥2. A severe migraine day was defined as a day with VAS score ≥ 7.

Three mixed models were performed, each of them using one of the three output measures as a dependent variable. The independent variables were time (baseline, W1, W2) and brain stimulation condition (prefrontal, occipital, or sham). The models were built using the lme() function in R with the patient ID being considered as random effect while also assessing the interaction effect between the brain stimulation condition and time as fixed effect. In the mixed-effect models, *p* values are based on the conditional F-tests and adjustment for degrees of freedom using the Kenward–Roger approximation (the pbkrtest-package). Data are reported as mean ± standard error. For all tests, statistical significance was set at 0.05.

## 3. Results

**Figure 2:**
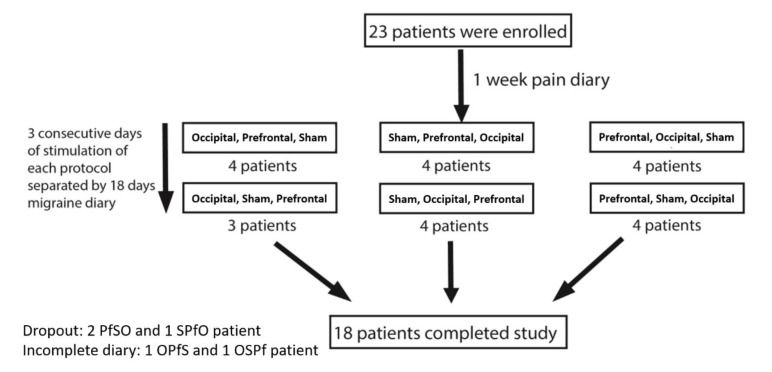
Flowchart of the study. S, Pf, and O refer to sham, prefrontal, and occipital blocks, respectively.

Three patients dropped out of the study: two after undergoing the prefrontal condition and one following the sham condition. Two additional patients were excluded because of incomplete migraine diaries. Withdrawal related to side effects did not occur. In total, 18 patients successfully completed the study. The flowchart of the study is presented in Figure 2. Baseline demographics and clinical data are summarized in Table 1.

**Figure 3:**
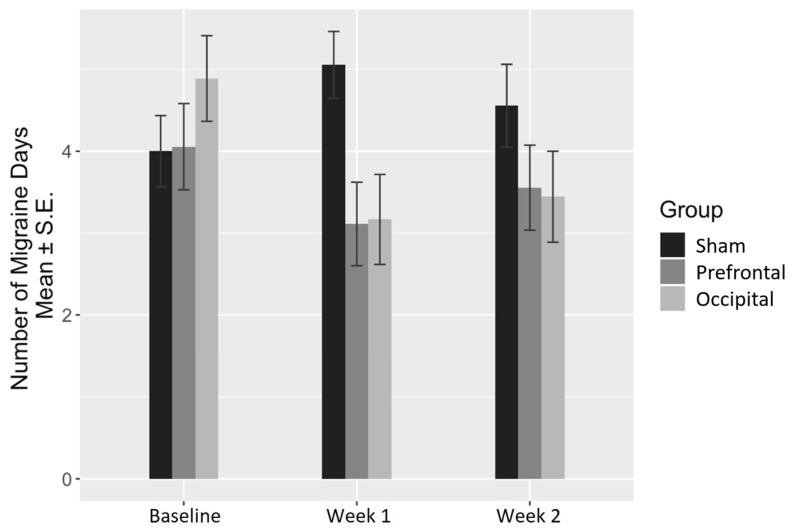
Change in the number of migraine days per week.

Concerning the number of migraine days, the brain stimulation condition and the interaction between the brain stimulation condition and time were found to be significantly associated with the number of migraine days per week (ANOVA *p* = 0.007 and *p* = 0.006, respectively). More specifically, the interaction between occipital stimulation and time was found to be significant at both W1 (coefficient = −2.78, *p* < 0.001) and W2 (coefficient = −2.00, *p* = 0.010). However, prefrontal stimulation was found to have a significant interaction with time at W1 only (coefficient = −2.00, *p* = 0.010) (Table 2). Descriptive data according to time and stimulation condition are summarized in Table 3 and illustrated in Figure 3.

With respect to the number of severe migraine days, brain stimulation condition and its interaction with time were found to be significantly associated with the number of severe migraine days (ANOVA *p* < 0.0001 and *p* = 0.017, respectively). The interaction between occipital stimulation and time was found to be significant at both W1 (coefficient = −2.44, *p* = 0.001) and W2 (coefficient = −1.61, *p* = 0.029). Whereas prefrontal stimulation was found to have a significant interaction with time at W1 only (coefficient = −1.72, *p* = 0.019). 

In addition to these interactions, the independent effect of occipital stimulation was found to be significantly associated with the number of severe migraine days per week (coefficient = 3.78, *p* < 0.001) (Table 4). 

**Figure 4:**
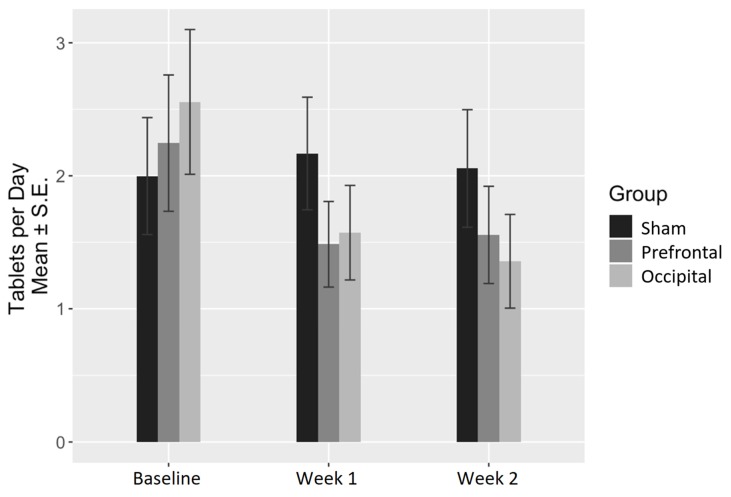
Change in the number of consumed pills per day.

Regarding pills consumption, the time factor was the most significantly associated with the number of consumed pills per day (ANOVA *p* value = 0.007). In fact, the only significant interaction between the stimulation condition and time was for the occipital stimulation at both W1 (coefficient = −1.15, *p* = 0.023) and W2 (coefficient = −1.26, *p* = 0.014) (Table 5). No significant interaction between prefrontal stimulation and time was found, neither at W1 nor at W2. Descriptive data according to time and stimulation condition are summarized in Table 6 and illustrated in Figure 4.

## 4. Discussion

The results of the current work support a significant benefit of cathodal tDCS of the occipital cortex and anodal tDCS of the prefrontal cortex in patients with MOH. Whereas both conditions resulted in a reduction of the total number of migraine days and severe migraine days in the week following the intervention, only occipital tDCS resulted in a long-lasting effect extending to the end of the follow-up period (14 days in total). Furthermore, a reduction in pills consumption was only observed with occipital tDCS, this effect also lasted for two weeks after the last stimulation session. The procedure was well tolerated, and no serious side effects were recorded, making it a safe non-pharmacological option. The results of this pilot study in MOH are preliminary and merit confirmation in large-scale, randomized clinical trials.

Treatment options in MOH are limited and poorly supported by good-quality data. The standard treatment has long been early withdrawal of the overused medication [32]. When this is unsuccessful because of intolerable rebound headaches, alternative strategies have been proposed such as in-patient withdrawal and bridging therapy with prednisone [1]. Whatever the approach, the outcome seems to be better if a migraine prophylactic treatment is added to early discontinuation [1]. Alternatively, prophylactic treatment without withdrawal of the overused medication can reduce the frequency of headaches and acute medication consumption. The present study lends further support to this approach and suggests that deliberate withdrawal of the overused medication is not always necessary, which could help avoid rebound headaches that often cause significant patient distress. Besides its positive impact on the total number of migraine days, occipital tDCS also resulted in a significant decrease in the number of severe migraine attacks up to 2 weeks following the procedure. This would explain the lasting effect of tDCS on the need for abortive medications.

This proof of concept study has many limitations and was not designed to answer many clinically relevant questions. Although pills consumption was significantly decreased during the 2-week follow-up period, most patients were still consuming analgesics. It is important to highlight that these results were observed after only three tDCS sessions, and it is tempting to believe that increasing this number would result in more pronounced and sustained effects, but this remains to be proven in future studies with a longer follow-up period. In addition, it would be interesting to see whether an optimized tDCS protocol with regular maintenance sessions can lead to complete withdrawal of medications, and whether combining tDCS with prophylactic or bridging therapy could further improve the outcome.

On a different note, the results of the present work suggest tDCS efficacy in preventing migraine in the context of MOH. However, the mechanisms underlying these effects remain to be explored. Regarding occipital tDCS, it is tempting to believe this condition helped correct the physiological abnormalities within the occipital cortex (e.g., cortical hyperexcitability) and/or its connections observed in migraineurs [21,22,23,24,25,26,27,28,29]. As for the prefrontal tDCS, several mechanisms are plausible such as an increase in the top-down inhibitory control of pain, modulation of the emotional aspect of pain, or suppression of substance-seeking behavior [26,30,31,32,33].

The effects of tDCS on other comorbidities (i.e., depression and anxiety symptoms) were not specifically studied. This would have been relevant since a dynamic bidirectional relationship seems to exist between migraine and anxiety and depression [27]; the latter symptoms seem to correlate with the frequency of headache attacks as well as the occipital hyperexcitability state described in migraine [27]. Moreover, the anodal stimulation of the prefrontal cortex is an established treatment for depression, which provides an additional reason to account for psychiatric symptoms in migraine studies [9]. Another drawback of this study comes from the lack of allodynia scores. Indeed, allodynia has been widely described among migraineurs, seems to reflect a hyperactivation of the trigeminal system, and has been linked to an increase in headache frequency and development of chronic headache condition [28]. Therefore, it cannot be excluded that some of the benefits observed in this study were related to improvement of these comorbid conditions. Hence, upcoming tDCS studies would benefit from including measures for allodynia and affective symptoms, which would enable an understanding of how each symptom impacts the clinical response.

In addition, it would have been interesting to investigate the existence of site-specific effects on the different subtypes of MOH. The small size of this study did not allow such an investigation, which is particularly relevant to patients overusing medications containing psychoactive substances; a situation in which the prefrontal stimulation site could theoretically have an advantage over the occipital one.

Furthermore, it is important to take into consideration the employed tDCS setups. For instance, in the case of occipital tDCS, the reference electrode was over the contralateral supraorbital area as in previous studies [19]. Other migraine studies targeting the occipital cortex have selected another cephalic reference (over Cz in [11]) or an extracephalic one (i.e., over the chin in [14]). In order to determine the optimal montage, it would be relevant to simulate the current flow and/or compare the clinical outcomes obtained in the various setups. Beside the position of the return electrode, increasing the spatial focality of tDCS would enable an understanding of the neural substrates at the basis of the observed clinical response and might optimize the outcomes. This could be obtained by reducing the electrode size, using ring electrodes versus the conventional rectangular pad, or employing high-definition tDCS (i.e., one central electrode surrounded by four reference electrodes) [34,35].

Lastly, an additional limitation of this work is related to the short-term follow-up. Future large-scale studies are required in order to assess the long-term effects of such interventions, define the clinical profile of responders, and explore the potential impact of tDCS on migraine-associated symptoms.

## 5. Conclusions

tDCS seems to have beneficial effects in patients with MOH. The wide availability, safety profile, tolerability, practicality, relatively low cost, and few contraindications of this non-invasive brain stimulation technique render it particularly interesting in specific populations for whom medical treatments are contraindicated (i.e., presence of comorbidities) or limited to very few options (i.e., in case of pregnancy and lactation) [36]. Large-scale randomized clinical trials are required in order to establish the exact role of tDCS in managing MOH.

## Figures and Tables

**Table 1:** Demographic and clinical characteristics displayed as number of patients unless specified otherwise.

Sociodemographic and Clinical Variables	Results
Mean age in years (range)	40.3 (20–38)
Gender	
Male	1
Female	17
Side of pain	
One side	14
Bilateral	4
Male with aura	0
Male without aura	1
Female with aura	7
Female without aura	10
Positive family history Prophylactic medications Topiramate Amitriptyline	12 1 4
Abortive medications	
NSAIDs	7
Paracetamol	5
Paracetamol codeine	1
Combination of above	5
Using OCPs	0

NSAIDs: non-steroidal anti-inflammatory drugs; OCPs: oral contraceptive pills.

**Table 2:** Mixed-effect model for the number of migraine days with the ‘patient ID’ being considered as random effect and the interaction between the brain stimulation condition and time as fixed effect.

Predictors	Value		
	Estimates	CI	*p* Value
(Intercept)	4.00	3.00; 5.00	**<0.001**
Prefrontal	0.06	−1.01; 1.12	0.918
Occipital	0.89	−0.18; 1.95	0.101
Week 1	1.06	−0.01; 2.12	0.052
Week 2	0.56	−0.51; 1.62	0.304
Prefrontal × Week 1	−2.00	−3.51; −0.49	**0.010**
Occipital × Week 1	−2.78	−4.28; −1.27	**<0.001**
Prefrontal × Week 2	−1.06	−2.56; 0.45	0.168
Occipital × Week 2	−2.00	−3.51; −0.49	**0.010**

Significant *p* values are bolded.

**Table 3:** Summary statistics of the number of migraine days per week in the three brain stimulation conditions at baseline, week 1, and week 2.

	Baseline	Week 1	Week 2
Sham	4.00 ± 0.44	5.06 ± 0.41	4.56 ± 0.51
Prefrontal	4.06 ± 0.53	3.11 ± 0.51	3.56 ± 0.52
Occipital	4.89 ± 0.52	3.17 ± 0.55	3.44 ± 0.56

Data are represented as mean ± standard error.

**Table 4:** Mixed-effect model for the number of severe migraine days per week with the ‘patient ID’ being considered as random effect and the interaction between the brain stimulation condition and time as fixed effect.

Predictors	Value		
	Estimates	CI	*p* Value
(Intercept)	1.11	0.20; 2.03	**0.018**
Prefrontal	0.72	−0.30; 1.74	0.163
Occipital	3.78	2.76; 4.80	**<0.001**
Week 1	0.72	−0.30; 1.74	0.163
Week 2	0.17	−0.85; 1.18	0.747
Prefrontal × Week 1	−1.72	−3.16; −0.28	**0.019**
Occipital × Week 1	−2.44	−3.88; −1.00	**0.001**
Prefrontal × Week 2	−0.83	−2.27; 0.61	0.254
Occipital × Week 2	−1.61	−3.05; −0.17	**0.029**

Significant *p* values are bolded.

**Table 5:** Mixed-effect model for the number of consumed tablets per days with the ‘patient ID’ being considered as random effect and the interaction between the brain stimulation condition and time as fixed effect.

Predictors	Value		
	Estimates	CI	*p* Value
(Intercept)	2.00	1.16; 2.84	**<0.001**
Prefrontal	0.25	−0.45; 0.95	0.486
Occipital	0.56	−0.14; 1.26	0.118
Week 1	0.17	−0.53; 0.87	0.633
Week 2	0.06	−0.64; 0.76	0.872
Prefrontal × Week 1	−0.93	−1.92; 0.06	0.066
Occipital × Week 1	−1.15	−2.15; −0.16	**0.023**
Prefrontal × Week 2	−0.75	−1.74; 0.25	0.139
Occipital × Week 2	−1.26	−2.25; −0.26	**0.014**

Significant *p* values are bolded.

**Table 6:** Summary statistics of the number of consumed pills per day in the three brain stimulation conditions at baseline, week 1, and week 2. Data are represented as mean ± standard error.

	Baseline	Week 1	Week 2
Sham	2.00 ± 0.44	2.17 ± 0.42	2.06 ± 0.44
Prefrontal	2.25 ± 0.51	1.48 ± 0.32	1.56 ± 0.37
Occipital	2.56 ± 0.54	1.57 ± 0.36	1.36 ± 0.35
